# A Review and Report of Peripheral Giant Cell Granuloma in a 4-Year-Old Child

**DOI:** 10.1155/2016/7536304

**Published:** 2016-06-15

**Authors:** Afsaneh Nekouei, Alireza Eshghi, Parisa Jafarnejadi, Zahra Enshaei

**Affiliations:** Torabinejad Dental Research Center and Department of Pediatric Dentistry, School of Dentistry, Isfahan University of Medical Sciences, Isfahan 73461-81746, Iran

## Abstract

Peripheral giant cell granuloma is a common benign and reactive gingival epulis in oral cavity. It is often difficult to make a clinical diagnosis; thereby definitive diagnosis depends on histopathologic features. We report a case of a 4-year-old Caucasian boy presenting with a five-month history a 20 × 15 × 12 mm pedunculated, lobular soft tissue mass of the left anterior maxilla gingiva which was misdiagnosed and maltreated before his referral. An excisional biopsy of the lesion followed by histopathologic examination of the biopsy specimen revealed distinctive features of peripheral giant cell granuloma. Early detection and excision of this hyperplastic nodule especially in children are important to minimize potential dentoalveolar complications.

## 1. Introduction

Solitary gingival enlargements in children are relatively common and usually occur in reaction to local irritation or chronic trauma. One of these enlargements is peripheral giant cell granuloma (PGCG), a lesion unique to the oral cavity, occurring only on the gingiva. It is distinguishable from similar lesion only on the basis of its unique histomorphology, which is essentially identical to that of central giant cell granuloma, intrabony benign neoplasm of the jawbone [1].

Peripheral giant cell granuloma originates from the interdental tissues (periosteum or periodontal membrane) [2, 3]. Lesions can arise anywhere on the gingival or alveolar mucosa but most occur anterior to the molar teeth [2, 4]. Although these lesions occur over a wide age range, the peak incidence in males is in the second decade compared to the fifth decade for females [5]. There is a nearly 2 : 1 predilection of females to males, with the mandible being involved more often than the maxilla [6]. While incipient lesions often present as painless, lobular, and ulcerated masses with little complications and minor changes in gingival contour, progressive growth in some cases causes a significant swelling interfering normal oral function and resorption of the subjacent alveolar bone and teeth roots [2].

Our study is aimed to report a case of PGCG in a young child which was misdiagnosed and neglected for more than 5 months. We review histopathologic features and discuss possible differential diagnosis, based on the age of the patient, history, and clinical features.

## 2. Case Report

A 4-year-old Caucasian boy was referred from a private office to our pediatric dentistry clinic with a complaint of swelling in left anterior maxillary canine and lateral incisor teeth area. It was started about five months previously bilaterally in a smaller dimension. The right lesion subsided without any intervention but the left lesion has been enlarging with parents and child manipulation. The central and lateral primary incisors were extracted 3 months ago by a general dentist with the diagnosis of periapical abscess but the lesion did not heal and has been progressively growing. The lesion was painless and with no associated spontaneous bleeding except for occasional interference of the swelling with mastication. His medical history includes no complication.

**Figure 1:**
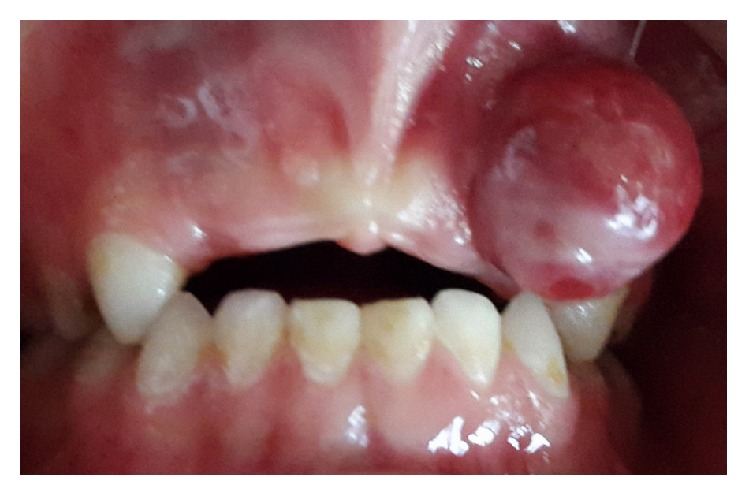
Intraoral preoperative view of lesion: a painless red enlargement of the maxillary attached gingiva extends to the alveolar mucosa between teeth #52 and 53.

**Figure 2:**
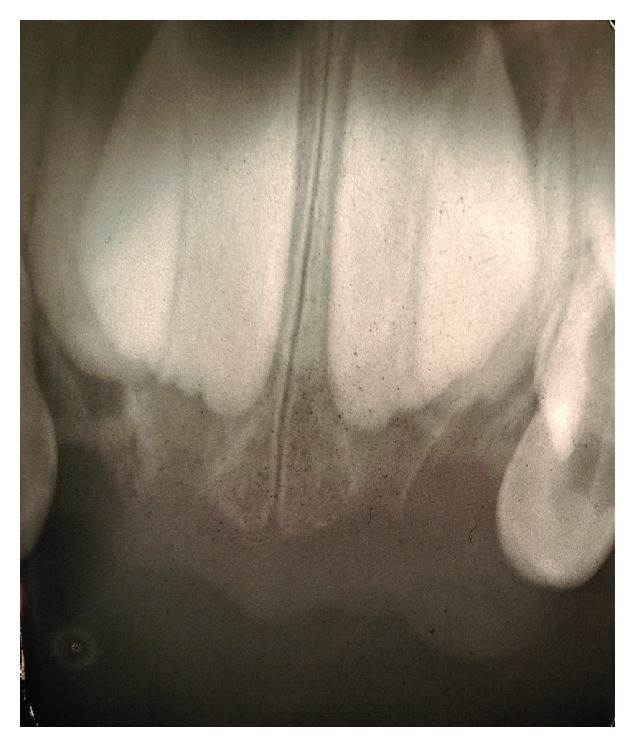
Intraoral periapical radiograph, showing superficial erosion of the alveolar bone.

On clinical examination, he had slight extraoral facial swelling of his left anterior maxilla, without any palpable regional lymph nodes. An intraoral examination showed a 20 × 15 × 12 mm pedunculated, lobular soft tissue mass of his left anterior maxillary gingiva related to his incisors (Figure 1). On palpation, the lesion had a firm consistency. The mucosal covering of the lesion exhibited surface tan, red, and bluish areas with a focal area of ulceration. A periapical radiograph of this area revealed superficial erosion of the alveolar bone with no other significant findings (Figure 2).

These clinical and radiographic findings indicated a benign lesion, and the following differential diagnoses were considered: parulis, pyogenic granuloma, peripheral ossifying fibroma, PGCG, and peripheral odontogenic fibroma. Malignant entities such as squamous cell carcinoma, other primary malignant lesions, and metastatic lesions, although thought to be unlikely, were also considered.

**Figure 3:**
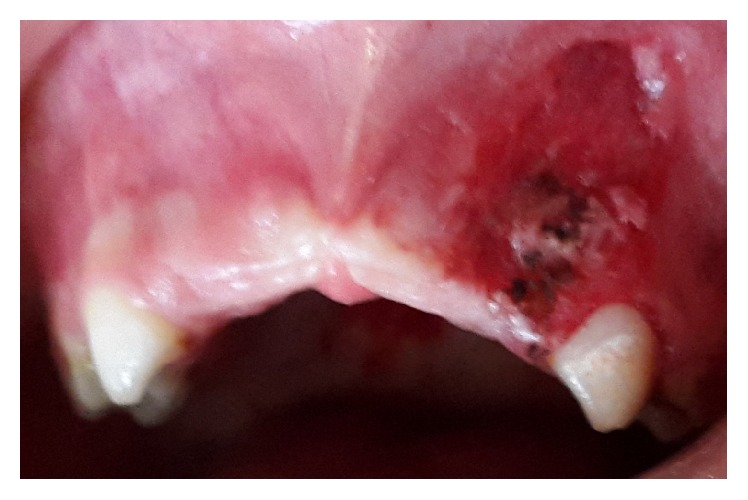
Intraoral immediate postoperative view showing complete surgical removal.

The patient was scheduled for surgical excision. Under local anaesthesia, the lesion was excised down to the periosteum (Figure 3). After complete excision of the lesion, the exposed surface was cauterized to control the bleeding and the entire specimen submitted for histopathologic examination.

**Figure 4:**
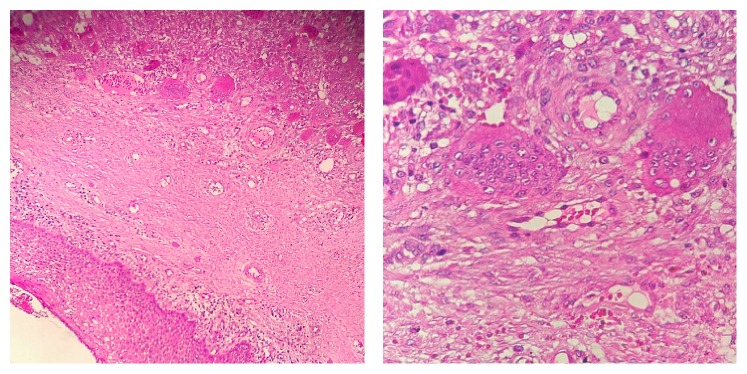
Histopathologic section: hyperplastic granulation tissue, the presence of acute and chronic inflammatory cells, capillaries, and proliferation of multinucleated giant cells within haemorrhagic background showing histological appearance of the PGCG lesion (H&E stain 10x and 40x).

Microscopic examination shows multinucleated giant cell proliferation within a background of spindle-shaped and ovoid mesenchymal cells. Areas of haemorrhage and acute and chronic inflammatory cells are frequently present. A zone of dense fibrous connective tissue separates giant cell proliferation from mucosal surface. Areas of dystrophic calcifications and reactive bone formation are seen around (Figure 4). This histopathology confirmed the entity of PGCG.

Follow-up visits were scheduled at three week intervals. No signs of recurrence of the lesion have been observed during nine months after the excision.

## 3. Discussion

While PGCG occurs mostly in adults, some cases have been described in children where a more aggressive clinical behaviour has been observed [7]. In a review of 720 cases, 33% were seen in patients younger than 20 years of age, which concurs with the findings of another study in which 33 of 97 cases (34%) occurred in individuals between 5 and 15 years of age [8, 9]. Studies show the clinical features of peripheral giant cell granuloma in Iranian population are almost similar to those reported by other investigators with age ranged from 6 to 75 years (mean 33 years) [10]. The case we reported was 4 years old and below minimum threshold for PGCG common age range.

In children, PGCG similar to other reactive oral lesions appears to have a rapid growth rate, be more aggressive with infiltration of the interproximal crest area and bone resorption, interfere with eruption of adjacent teeth, produce minor to moderate tooth movement, and have multiple recurrences [11]. Therefore considering the probability of PGCG in gingival enlargements even in the children under 5 years old reduces its consequences and prevents clinicians' misdiagnosis as in our case that lead to unreasonable extraction of primary incisors. PGCG can be easily distinguished from parulis, which is frequently associated with a necrotic tooth or with periodontal disorder. Radiographs are essential for confirming the oral mucosa origin of the giant cell lesion and refusing a central bony lesion with cortical perforation and soft tissue extension. Early detection of the PGCG results in more conservative surgery with less risk for tooth and bone loss [2].

In differential diagnosis in the cases of gingival enlargements in children we consider four main lesions: pyogenic granuloma, peripheral giant cell granuloma, peripheral ossifying fibroma, and peripheral odontogenic fibroma. Peripheral giant cell granuloma, like the peripheral ossifying fibroma, is a lesion unique to the oral cavity, occurring only on the gingiva. Unlike peripheral ossifying fibroma, however, it may occur on the alveolar mucosa of edentulous areas. Like pyogenic granuloma and peripheral ossifying fibroma, peripheral giant cell granuloma may represent an unusual response to tissue injury. It is distinguishable from pyogenic granuloma and peripheral ossifying fibroma only on the basis of its unique histomorphology, which is the same as central giant cell granuloma [8, 9]. Clinically, peripheral odontogenic fibroma (WHO type) must be considered in the differential diagnosis of dome-shaped or nodular, nonulcerated, growths on the gingiva like PGCG. Peripheral odontogenic fibroma is characterized by a fibrous or fibromyxomatous stroma containing varying numbers of islands and strands of odontogenic epithelium that is clearly distinguishable from PGCG histopathology [12].

Clinically, PGCG features separate it from the fibrous and vascular epulides. It presents as a firm, soft, bright pedunculated or sessile nodule with various sizes that range from small papules to enlarged masses, though they are generally less than 20 mm in diameter with the color ranging from dark red to purple or blue commonly with ulcerated surface [3, 13]. Pain is not a common characteristic, and lesion growth in most cases is induced by repeated trauma [1].

The peripheral giant cell granuloma is best treated by complete surgical excision, with care taken to excise it at its base [14]. The treatment of PGCG comprises surgical resection with elimination of the entire base of the lesion in addition to the eradication of the underlying source of irritant factors [2, 3]. If incomplete bone resection was done, the growth may recur [2]. To avoid recurrence after treatment, in addition to complete simple excision with extensive clearing of the base of the lesion, the source of irritation needs to be removed [4].

Recurrence of PGCG is not common and ranges as little as 5–11% [15, 16] but multiple recurrences with eventual loss of the adjacent teeth are a potential complication [2]. Early diagnosis based on clinical and radiological findings, confirmed by pathological analysis especially in children, allows for conservative management with less risk of destruction for the adjacent teeth and tissues.

## 4. Conclusion

Peripheral giant cell granuloma as relatively common lesion should be considered in the cases of gingival enlargements in children. Histopathology is the diagnostic tool for ejecting similar lesions. Surgical excision is a successful treatment of choice in minimizing the recurrence of lesion. Regardless of the surgical technique employed, it is important to eliminate the etiological factors and to examine the tissues histologically for confirmation. Hence, the consideration should also be given to correct diagnosis and proper treatment planning.
